# Perioperative dynamic alterations in peripheral regulatory T and B cells in patients with hepatocellular carcinoma

**DOI:** 10.1186/1479-5876-10-14

**Published:** 2012-01-25

**Authors:** Tianxiang Chen, Dongli Song, Zhihui Min, Xiangdong Wang, Yu Gu, Bajin Wei, Jia Yao, Kangjie Chen, Zhijun Jiang, Haiyang Xie, Lin Zhou, Shusen Zheng

**Affiliations:** 1 Division of Hepatobiliary and Pancreatic Surgery, Department of Surgery ,First Affiliated Hospital, School of Medicine, Zhejiang University, Hangzhou, China; 2 Key Laboratory of Combined Multi-organ Transplantation, Ministry of Public Health, Key Laboratory of Organ Transplantation, Zhejiang Province, Hangzhou, China; 3 Biomedical Research Center, Zhongshan Hospital, Fudan University, Shanghai, China; 4 Department of Surgical Oncology, First Affiliated Hospital, School of Medicine, Zhejiang University, Hangzhou, China

**Keywords:** regulatory T cells, regulatory B cells, hepatocellular carcinoma, surgery, dynamic alteration, lymphocytes, clinical informatics

## Abstract

**Background:**

Intratumoral and circulating regulatory T cells (Tregs) have been shown to be critical in the pathogenesis of hepatocellular carcinoma (HCC). However there is limited knowledge on the alterations of regulatory B cells (Bregs). We here investigated perioperative dynamic alterations of peripheral circulating Tregs and Bregs in HCC patients to reveal the relationship between regulatory lymphocytes and its clinical implications.

**Methods:**

36 patients with HCC, 6 with chronic hepatitis B infection and 10 healthy donors were enrolled for this study. Frequencies of peripheral Tregs and Bregs were measured by flow cytometry with antibodies against CD4, CD25, CD127, CD19 and IL-10 before, and after radical surgery. Then, clinical informatics of HCC patients was achieved through Digital Evaluation Score System (DESS) for the assessment of disease severity. Finally, we analysed correlations between digitalized clinical features and kinetics of circulating regulatory lymphocytes.

**Results:**

Level of circulating CD4^+^CD25^+^CD127^- ^Tregs in HCC patients was significantly lower than that in healthy donors and patients with chronic hepatitis B infection before surgery, but was increased after surgery. Preoperative level of CD19^+ ^IL-10^+ ^Bregs in HCC patients was also significantly lower than the other groups. However it dramatically was elevated right after surgery and remained elevated compared to controls (about 7 days after surgery, *P *= 0.04). Frequency of circulating Tregs was correlated with circulating leukocytes, ferritin, and clinical features suggesting tumor aggressiveness including portal vein thrombosis, hepatic vein involvement and advanced clinical stages. Frequency of circulating Bregs was associated with Hepatitis B e Antigen (HBeAg) and Hepatitis B virus (HBV) DNA copy number. In addition, DESS was significantly and positively correlated with other staging systems.

**Conclusion:**

Frequencies of peripheral Tregs and Bregs in HCC patients increased after surgery. These results suggest that a postoperative combination of therapies against Tregs and Bregs may be beneficial for better outcome of HCC patients after resection.

## Background

Regulatory T cells (Tregs) are a subpopulation of CD4^+ ^and CD8^+ ^T cells with immune suppressive function. In cancer patients especially patients with hepatocellular carcinoma (HCC), Tregs contribute to the dampening of the antitumor immune response [1,2]. Patients undergoing hepatic resection for HCC with prominent Treg infiltration showed increased recurrence and worse prognosis. Intratumoral Tregs have further been proposed to be an independent prognostic factor in HCC patients by several publications. In combination with cytotoxic T cells, Tregs can predict prognosis more effectively [3,4]. In addition, increased CD4^+^CD25^+ ^Tregs in the tumor microenvironment of HCC were found to be correlated with tumor size and vascular invasion [3-5]. On the other hand, Ormandy and others first reported peripheral CD4^+^CD25^+^Tregs were increased in HCC patients [6]. However, contradict results were also described by others [7].

Recently, regulatory B cells (Bregs), a new family of regulatory cells, were found to control immune responses at both innate and adaptive levels [8,9]. Expansion of Bregs was demonstrated to inhibit harmful immune responses in chronic inflammation by deactivation of effector T cells and natural Killer T cells [10,11]. Furthermore, the suppressive immune function of Bregs appears to be in contact-dependent and independent manner [12]. These immune regulatory mechanisms comprise of protection from lethal inflammation, modulation of the development of autoimmune diseases [13-15], and inhibition of anti-cancer response in various tumor models [9,16-18]. However, few studies assess the role of Bregs in HCC development.

Although compelling evidence has suggested the important roles of both Tregs and Bregs in tumor development, few researches described both of them together in HCC patient samples. In the present study, we investigated perioperative alterations of both circulating Tregs and Bregs in patients with HCC and their relations to clinical phenotypes were examined. Clinical phenotypes, as clinical informatics, were achieved by a Digital Evaluation Score System (DESS) for assessing the severity of patients [19]. Frequencies of both circulating Tregs and Bregs elevated after surgery. These results suggest that a combined deletion of both Tregs and Bregs may be essential for better prognosis of patients with HCC after surgery. We also found significant correlations between digitalized clinical features and both peripheral regulatory lymphocytes. Integration of clinical informatics and experimental results is a useful method to conduct translational research.

## Methods

### Patient Population

Of 230 candidates from outpatients and inpatients, the current case-controlled study recruited 36 patients with primary liver cancer, 6 with chronic Hepatitis B infection (CHB) as chronic liver disease control, and 10 healthy donors as normal controls. None of patients with liver cancer received any invasive treatments like transcatheter arterial chemoembolisation or radiation frequency ablation before tumor resection. Among patients with liver cancer, 32 were pathologically diagnosed as hepatocellular carcinoma, while 4 as combination of hepatocellular and cholangiocarcinoma. Additionally, patients with chronic HBV infection enrolled were Hepatitis B Surface Antigen-positive for greater than 6 months according to its definition [20]. Based on clinicapathological features, patients were divided into different clinical stages by tumor node metastasis (TNM) staging system [21]. This project was approved by the Ethical Committee of First Affiliated Hospital of School of Medicine of Zhejiang University (NO.2011-108) and conducted in compliance with the Helsinki Declaration. All the participants were explained the investigative nature of the study and signed an informed consent before entry into study.

### Samples collection and preparation

Peripheral blood samples were intravenously drew and collected. Samples were collected immediately after admission before any intervention, 1-2 days and about 7 (5-9) days after tumor resection. Samples of patients with chronic HBV infection were collected when patient were just admitted without therapeutic intervention. Peripheral blood mononuclear cells (PBMC) were isolated according to previously study [22,23]. In brief, whole blood samples were overlaid onto Ficoll separation media (Shanghai Danfan Network Science & Technology, Shanghai, China) after 1:1 dilution with Hank's Balanced Salted Solution (Gibco, CA, USA). PBMC were centrifuged for 20 min at X1900 rpm, collected at the plasma interface and washed thrice after centrifugation at X1000 rpm for 10 min. PBMC were frozen and stored in liquid nitrogen till flow cytometry analysis.

### Flow cytometry analysis

Flow cytometry analysis was conducted by FACS Aria II flow cytometer (BD Bioscience, San Diego, CA, USA). For surface staining, suspensions of PBMC were stained on ice using predetermined optimal concentrations of each antibody for 30 min, and fixed using fixation buffer (BD PharMingen, San Diego, CA). Tregs identified with CD4^+^CD25^+^CD127^- ^expression were stained with human regulatory T cell Cocktail (BD PharMingen) [24] and Bregs identified with IL10^+^CD19^+ ^expression were stained with human anti-CD19 PerCp-Cy5.5 and Human anti-IL10 PE (BD PharMingen) [25]. Intracellular IL-10 analysis was performed by flow cytometry, as described previously [25,26]. Briefly, cells were resuspended (2 × 10^6 ^cells/ml) in medium and stimulated with CpG (Invivogen, San Diego, CA) and CD40L (1 μg/ml; R&D Systems, Minneapolis, MN), Phorbol-12-myristate-13-acetate (PMA,50 ng/ml; Sigma), ionomycin (1 μg/ml; Sigma), Brefeldin A (1X solution/ml; BD Bioscience) right before staining and flow cytometry analysis. After surface staining, for IL-10 detection, Fc receptors were blocked using FcR-Binding inhibitor (eBioscience, San Diego, CA). Cells were fixed, permeabilized using a Cytofix/Cytoperm kit (BD PharMingen), and stained with monoantibody against IL-10 according to the manufacturer's instructions. Results are expressed as the frequency of Tregs or Bregs.

### Digital Evaluation Score System for Liver Cancer

Digital Evaluation Score System (DESS) for patients with HCC was designed and implicated, as described previously with slight modifications [19]. DESS was established by senior surgeons who are specialized in hepatobiliary and pancreas disease to switch clinical pearls and materials of HCC patients into digitalized information. Briefly, DESS was integrated with descriptive information of patient history, signs, physical examination, clinical imaging, and pathology and laboratory tests. Of them, 165 clinical variables selected from HCC patients were included in DESS and divided into different sections such as history (Additional file 1 Table S1), signs and physical examination (Additional file 2, Table S2), combined laboratory test (Additional file 3 Table S3), imaging (Additional file 4 Table S4) and pathology (Additional file 5 Table S5). Severity of each variable was scored and calculated as 0, 1, 2 and 4 (Additional file 1, 2, 3, 4). The maximal value of score 4 means far more above physiological range or much more critical condition, while the minimal value of score 0 indicates the variable is within physiological range. Several variables were 0 or 4 like "fatigue", "enlargement of lymph nodes" and "goblin", because they are either lack of standard discrimination criteria or subdivision relies too much on patient's or physician's personal judgment. The value of 3 was specially excluded, since exponential values could better amplify distance among different severity levels [19]. Variables of laboratory tests in DESS were scored on basis of the results of preoperative measurements after patient admission without clinical treatment. After clinical data was transformed into points of each variable and put them together, the total score of DESS ranged from 0 to 660 points, higher scores in our design indicate a severer condition.

### Data analysis

All values were expressed as mean ± SEM. Statistical analysis was applied by SPSS software (SPSS 18.0; SPSS Inc; Chicago, IL). Frequencies of peripheral Tregs and Bregs among groups were analysed with one way ANOVA, followed by an unpaired student's *t*-test. Ranked data as single variable scores of DESS was compared by Mann-Whitney test. Correlations between DESS scores and frequencies of Tregs and Bregs and between the frequency of circulating total lymphocytes and that of Tregs and Bregs were performed by Spearman's rho test and Pearson's test as appropriate. *P *< 0.05 was considered as statistically significant.

## Results

### Perioperative alterations of peripheral Tregs and Bregs

**Figure 1:**
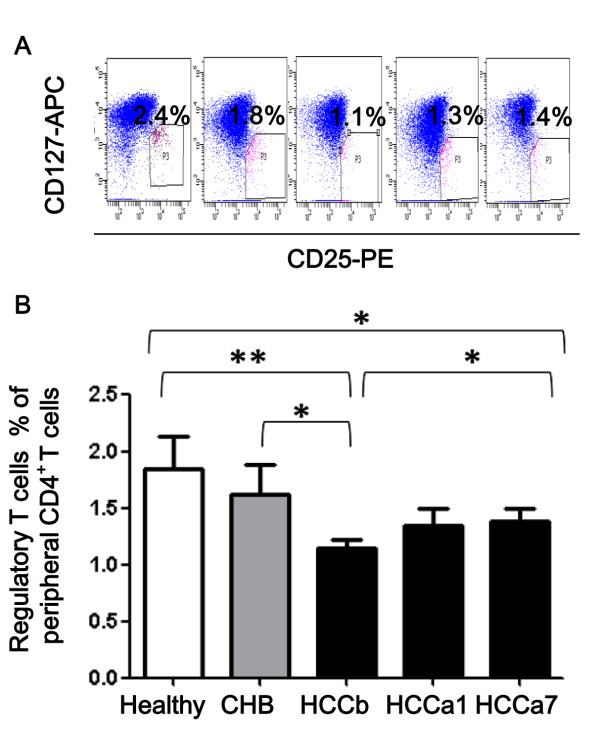
**Frequencies of peripheral regulatory T cells of different study groups**. Frequencies of peripheral regulatory T cells were analysed in the healthy donor (Healthy), patients with chronic hepatitis B infection (CHB), and patients with hepatocellular carcinoma (HCC) before surgery (HCCb), 1-2 days (HCCa1) or about 7 days (HCCa7) after surgery. Representative flow cytometry analysis of peripheral CD4^+^CD25^+^CD127^- ^populations were measured and presented (A). Frequency of CD4^+^CD25^+^CD127^- ^cells of total CD4^+ ^T cells in the peripheral blood were calculated (B). The mean ± SEM is shown,* and ** stand for *P *< 0.05 and 0.01, respectively.

Frequency of peripheral Tregs in HCC patients before surgery were significantly lower than that in the healthy (*P *= 0.002; Figure 1B) and CHB patients (*P *= 0.02; Figure 1B). 1-2 days after surgery, frequency of Tregs was not different to the original level (*P *= 0. 43). However, a significant elevation of frequency of Tregs was observed about 7 days after tumor resection, as compared with that before the operation (1.38 ± 0.12% in HCCa7 versus 1.14 ± 0.09% in HCCb, *P *= 0.04; Figure 1). Frequency of Tregs of HCC patients about 7 days after surgery was similar to that of patients with CHB (*P *= 0.19) though still lower than the healthy (*P *= 0.047).

**Figure 2:**
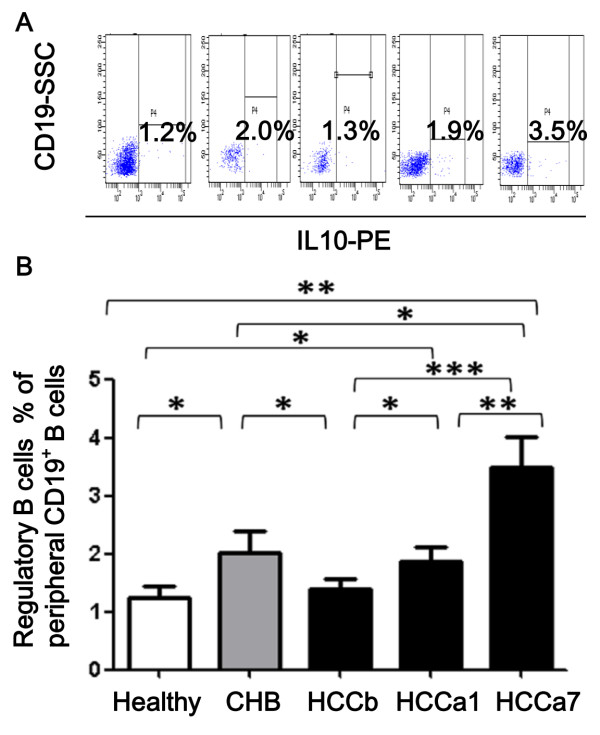
**Frequencies of peripheral regulatory B cells of different study groups**. Frequencies of peripheral regulatory B cells were analysed in the healthy donor (Healthy), patients with chronic hepatitis B infection (CHB), and patients with hepatocellular carcinoma (HCC) before surgery (HCCb), 1-2 days (HCCa1) or about 7 days (HCCa7) after surgery. Representative flow cytometry analysis of peripheral IL10^+^CD19^+ ^cells were measured and presented (A). Frequency of IL10^+^CD19^+ ^cells of total CD19^+ ^B cells in the peripheral blood (B). The mean ± SEM is shown, *, **and *** stand for *P *< 0.05, 0.01 and 0.001 respectively.

Frequencies of Bregs in the patients with CHB were significantly higher than those in the healthy and preoperative HCC patients (*P *< 0.05; Figure 2). Frequency of Bregs of HCC patients was significantly elevated by time after tumor resection (1.88 ± 0.23% in HCCa1 versus 3.48 ± 0.53% in HCCa7, *P *< 0.001; Figure 2), even significantly higher than that in CHB patients (HCCa7 versus CHB, *P *= 0.04; Figure 2).

### Circulating kinetics of lymphocytes with Tregs and Bregs

**Figure 3:**
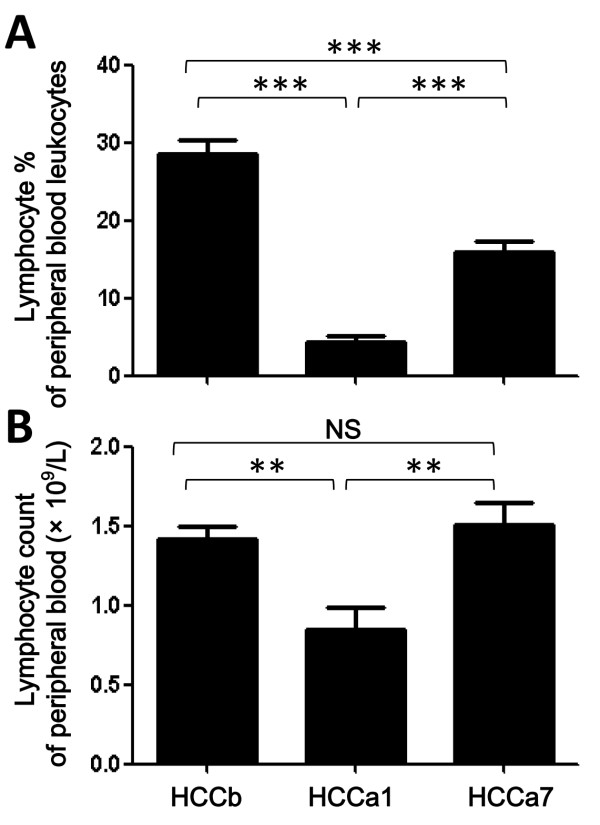
**Perioperative kinetics of peripheral lymphocytes of patients with hepatocellular carcinoma**. Frequency (A) and numbers (B) of lymphocytes in patients with hepatocellular carcinoma (HCC) before surgery (HCCb), 1-2 days (HCCa1) or about 7 days (HCCa7) after surgery. The mean ± SEM is shown, ** and *** stand for *P *< 0.01 and 0.001, respectively.

Frequency and number of circulating lymphocytes of HCC patients considerably declined 1-2 days after surgery, as compared with those before surgery (*P *< 0.01; Figure 3). However, it significantly elevated about 7 days after surgery, comparing with that 1-2 days after surgery (*P *< 0.001; Figure 3). The number of circulating lymphocytes about 7 days after surgery had no difference from the preoperative level. Frequency of circulating lymphocytes was correlated with the frequency of Tregs to some extent (*r *= 0.263, *P *= 0.028).

### Acquisition of General features of study population through DESS

All HCC patients (n = 36) were performed radical hepatic resection. Clinical informatics of HCC patients was digitalized through DESS as introduced in Methods. The mean value of HCC Patients' total score was 102.4 ± 3.9. Of them, 72% were stage I and the rest were more advanced. Various clinical features evaluated by DESS were compared between patients at mild stage (I) and advanced stage (II-IV). More than 10 features had higher DESS scores in patients at advanced stage (*P *< 0.05 or 0.01; Table 1). The mean rank of total scores of patients at advanced stage was also significantly higher than that at mild stage (24.3 in advanced stage group versus 16.3 in mild stage group, *P *= 0.04).

**Table 1:** Difference of clinical features between HCC patients at low and high stages of TNM (AJCC).

Variables	Mean Rank		*P*
	Stage Low	Stage High	
Platelet ×10^9^/L	16.77	23.00	0.034
Thrombocytocrit %	17.19	21.90	0.027
Cystatin C mg/L	16.08	24.80	0.003
Albumin/Globin	16.38	24.00	0.032
High density lipoprotein mmol/L	15.88	25.30	0.001
α-fetoprotein ng/ml	16.46	23.80	0.046
Ferritin ng/ml	15.62	26.00	0.004
Numbers of up-regulated tumor markers	15.48	26.35	0.003
Multiple Satellite nodules	16.77	23.00	0.053
Tumor encapsulation	16.88	22.70	0.022
score of Pathology section	15.58	26.10	0.007
Score of Tumor markers	16.35	24.10	0.042
Total score	16.27	24.30	0.040

Stage low: TNM (AJCC) stage I. Stage high: TNM (AJCC) stage II-IV.

HCC: Hepatocellular carcinoma; TNM (AJCC): Tumor-Node-Metastasis Staging System (American Joint Committee on Cancer).

### Correlation between DESS scores and other clinical staging systems

To better validate the value of DESS, patients were also staged using other 4 clinical staging systems: Liver Cancer Study Group of Japan/Tumor-Node-Metastasis Staging System(TNM/LCSGJ) [27], Child-Pugh Grading System [28], Barcelona Clinic Liver Cancer Staging System (BCLC) [29], and Cancer of Liver Italian Program Score (CLIP) [30]. The latter four systems are widely applied for the evaluation of liver cancer. Details of comparison between systems were summarized in Table 2. Significant and positive correlation between DESS and other staging systems was found (*P *< 0.05 or 0.01; Table 2). There was a significant and strongest correlation between BCLC and DESS (*r *= 0.501, *P *= 0.002), while not between Child-Pugh Grade and DESS.

**Table 2:** Correlations between total DESS scores and clinical stages of HCC patients.

	Total DESS scores	
Staging system	*r*	*P*
TNM (AJCC) stage	0.365	0.029
TNM (lCSGJ) stage	0.364	0.029
CLIP score	0.474	0.004
BCLC stage	0.501	0.002
Child-Pugh grade	-0.121	0.482

DESS: Digital evaluation scoring system; HCC: Hepatocellular carcinoma; TNM (AJCC): Tumor-Node-Metastasis Staging System (American Joint Committee on Cancer); TNM (LCSGJ): Tumor-Node-Metastasis Staging System (Liver Cancer Study Group of Japan); CLIP: Cancer of the Liver Italian Program Score; BCLC: Barcelona Clinic Liver Cancer Staging System.

### Correlation between clinical features and circulating Tregs and Bregs

Correlations of frequencies of peripheral Tregs and Bregs with DESS scores of clinical features were further examined in HCC patients (Table 3). High frequency of circulating Tregs was significantly correlated with the history of HBV infection (*P *= 0.031), circulating leukocytes (*P *= 0.029). The history of weight loss, plasma levels of ferritin, portal vein tumor thrombosis, hepatic vein invasion, and clinical stages evaluated by TMN systems or BCLC scores(*P <*0.05 or 0.01; Table 3) were inversely associated with frequency of circulating Tregs. At the same time, frequency of circulating Bregs has a positive correlation with HBeAg (*P *= 0.03) and HBV DNA copy number to some extent (*P *= 0.028).

**Table 3:** Correlations of clinical features of HCC patients with circulating Tregs and Bregs.

DESS evaluated	Tregs		Bregs	
Clinicopathological Characteristic	*r*	*P*	*r*	*P*
Weight Loss	-0.339	0.043*	-	-
HBV Infection History	0.360	0.031*	-	-
Leucocyte	0.363	0.029*	-	-
Hepatitis B e Antigen	-	-	0.527	0.030*
HBV DNA Copy number	-	-	0.532	0.028*
Ferritin	-0.451	0.006**	-	-
Numbers of up-regulated tumor markers ^#^	-0.376	0.024*	-	-
Portal vein tumor thrombosis	-.408*	0.014*	-	-
Hepatic vein invasion	-.413*	0.012*	-	-
BCLC score	-0.443	0.007**	-	-
TNM stage(AJCC)	-0.347	0.038*	-	-

(*, ** stands for *P *< 0.05, 0.01, respectively)

# Tested tumor markers:α-fetoprotein; cancer antigen 125; cancer antigen 153; cancer antigen 199; Ferritin; Carcino-embryonic antigen; prostate specific antigen.

HCC: Hepatocellular carcinoma; Tregs: Regulatory T cells; Bregs: Regulatory B cells; HBV: Hepatitis B virus; BCLC: Barcelona Clinic Liver Cancer Staging System; TNM (AJCC): Tumor-Node-Metastasis Staging System (American Joint Committee on Cancer).

## Discussion

Tregs, as a functionally unique subgroup of T lymphocytes, can suppress effective antitumor immune responses of various malignancies, including HCC [31,32]. The higher infiltration of Tregs in the local tumor microenvironment was found to correlate with poor prognosis of patients with HCC [3]. Likewise, increased frequency of peripheral Tregs was also shown to correlate with poor outcome [33]. However, unlike intratumoral Tregs, which were consistently reported to be increased in HCC patients, descriptions about frequency of peripheral Tregs were uncertain [5,6,34]. Since CD4^+^CD25^+^CD127^- ^was proved to be a classic surface marker for purified Tregs with highly suppressive function [35], Tregs were identified with CD4^+^CD25^+^CD127^- ^in the present study. Bregs are another special class of regulatory lymphocytes that modulating immune response. They have been suggested to be engaged in repressing autoimmune disease, chronic inflammatory diseases [13,14] and promoting tumor progression [16,18]. However, most current studies about Bregs were conducted in animal models and little was known on disease specific patterns of peripheral Bregs in HCC patients. In this study, we investigated the dynamic frequencies of both peripheral Tregs and Bregs among HCC patients during the perioperative period.

Our data showed that the peripheral frequency of Tregs in HCC patients before surgery was significantly lower than control groups, but increased after tumor resection. No statistical difference were found between the healthy and CHB patients. Therefore, it indicates that comparatively lower levels of peripheral Tregs in HCC patients may be associated with HCC itself, rather than the chronic inflammatory phase. One hypothesis forwarded includes recruitment of peripheral Tregs to the local environment[36]. The migration of peripheral Tregs in HCC patients has been suggested to be related to the production of chemokines from HCC [37].

The present results demonstrated that the peripheral frequency of Bregs was higher in CHB patients as compared with healthy donors and HCC patients. It is possible that the systemic inflammatory state caused by hepatitis induces the expansion of peripheral Bregs. Similarly, the frequency of Bregs was dramatically increased in HCC patients after surgery, with the level higher than CHB patients 7 days following resection. To our knowledge, we are the first to show the perioperative dynamic changes to circulating Tregs and Bregs in HCC patients and found a similarly increased pattern of them after radical surgery. Further studies are needed to clarify such elevation is resulted from the removal of tumor or extensive liver surgery.

HCC may produce abundant disease specific cytokines and chemoattractants including IL-8 [38] and CCL 20 [37], some of which are responsible for the "homecoming" signals to orient regulatory lymphocytes into the tumor. After surgery, Tregs and Bregs may be temporally stocked in the peripheral circulation due to the descent of tumor chemoattractants. Bregs play an important role in the prevention of inflammation, autoimmune response [39,40] and antitumor effects [17]. Hence, Bregs are probably involved in the local balance of immune tolerance and immune suppression in HCC like Tregs. To clarify the disease-specific dynamic profile of Tregs and Bregs in HCC, perioperative frequency of peripheral Tregs and Bregs in patients with pancreatic cancer was also measured. We found similar pattern but relatively altered degree of Tregs and Bregs in patients with pancreatic cancer (data unshown).

Accumulating evidences showed interaction between Tregs and Bregs in tumor microenvironment. For example, Bregs in the lung metastasis from breast cancer have been reported to induce conversion of resting CD4^+^T cells to Tregs to support metastatic growth. These tumor-evoked Bregs express constructively activated stat3 and B7-H1 [16]. In additions, Bregs are involved in anti-inflammatory process through the promotion of Tregs in HCC, like those reported in autoimmune disease [41]. Our previous work discovered that the expression of B7-H1 and IL10 was up-regulated in HCC tissues [42]. Similar interactions between Breg and Tregs mentioned above may exist in human HCC development as well. Collectively, the postoperative increase of peripheral Tregs and Bregs in HCC patients might worsen the host immune system due to their established immune suppressive capabilities after surgery. This immunopathological condition of body caused by regulatory lymphocytes will promote tumor metastasis and recurrence. This could also partially explain the unsatisfactory outcome of HCC patients who even received radical surgery. Strategies against Tregs have been proved to enhance anti-cancer immunity [43]. Elimination of Bregs have also been suggested to be useful in the clearance of established tumor[44]. Therefore, a comprehensive adjuvant immunotherapy targeting both Tregs and Bregs may be beneficial for improving prognosis of post-surgery HCC patients.

Total number and percentage of circulating lymphocytes were initially deceased after surgery, but recovered within a week. During this period, a rapid proliferation of lymphocytes may occur. It was found that stem cells including mesenchymal stem cells could proliferate after surgery. Various growth factors produced by stem cells like heme oxygenase-1 could stimulate the expansion of Tregs and Bregs [45].

The application of clinical informatics made it easier to analyse the correlation between extensive clinical phenotypes and frequencies of peripheral Tregs and Bregs. Ferritin released by melanoma was reported to induce IL10 production of lymphocytes and suppress immune responses [46]. We found serum ferrtin levels were correlated with circulating Tregs in HCC patients supporting previous studies. In addition, the negative correlation of Tregs with up-regulation of a panel of routinely tested 8 tumor markers(α-fetoprotein, cancer antigen 125, cancer antigen 153, cancer antigen 199, ferritin, carcino-embryonic antigen, prostate specific antigen) has been discovered. Intratumoral Tregs were found to be positively correlated with vascular invasion [3]. In contrast, our data further demonstrated that the frequency of peripheral Tregs was correlated with portal vein thrombosis and hepatic vein invasion, supporting its connection with HCC aggressiveness. We found frequency of peripheral Bregs was positively correlated with HBeAg and HBV DNA copy numbers. These indicate that active HBV infection may stimulate the accumulation of circulating Bregs. To further explore the mechanism associated with the interactions between Tregs and Bregs, mediators from both regulatory cells should be profiled and validated. Network biomarkers depicting protein-protein interactions within both regulatory cells should be investigated by the integration of knowledge on protein annotations, interaction, and signalling pathway [47,48]. Dynamic network biomarkers should be further correlated with clinical informatics, thereby to select not only disease specific but also disease stage specific biomarker [49]. DESS as a useful tool of clinical informatics can be modified leading to extensive clinical application in future [19].

## Conclusions

We report that frequencies of both peripheral Tregs and Bregs in HCC patients were decreased before surgery and significantly elevated after resection. These results suggest that a postoperative combined strategy targeting Tregs and Bregs could be beneficial for HCC patients to improve their prognosis. The correlations discovered between peripheral regulatory lymphocytes and clinical features through DESS set an example of clinical translational medicine.

## List of abbreviations

Tregs: Regulatory T cells; Bregs: Regulatory B cells; HCC: Hepatocellular carcinoma; DESS: Digital evaluation scoring system; HBeAg: Hepatitis B e Antigen; HBV: Hepatitis B virus; CHB: Chronic hepatitis B virus infection; PBMC: Peripheral blood mononuclear cells; TNM (AJCC): Tumor-Node-Metastasis Staging System (American Joint Committee on Cancer); TNM (LCSGJ): Tumor-Node-Metastasis Staging System (Liver Cancer Study Group of Japan); BCLC: Barcelona Clinic Liver Cancer Staging System; CLIP: Cancer of the Liver Italian Program Score.

## Competing interests

All authors declare that they have no competing interests.

## Authors' contributions

TC, DS, ZM, XW performed and participated in analysis of laboratory experiments data. TC, XW, LZ, HX, ZJ and SZ participated in the design of experiments. TC, YG, BW, JY, and KC acquired, preserved clinical samples and participated in clinical data analysis. LZ, HX, ZJ and SZ provided administrative support and funded experiments. TC, XW, YG, KC and SZ drafted the manuscript. All authors have contributed and approved the final manuscript.

## Supplementary Material

Additional file 1 **Table S1**. Variables and point values used in DESS for liver cancer patient (History). Click here for file

Additional file 2 **Table S2**. Variables and point values used in DESS for liver cancer patient (Signs and physical exams). Click here for file

Additional file 3 **Table S3**. Variables and point values used in DESS for liver cancer patient (Laboratory test). Click here for file

Additional file 4 **Table S4**. Variables and point values used in DESS for liver cancer patient (Imaging). Click here for file

Additional file 5 **Table S5**. Variables and point values used in DESS for liver cancer patient (Pathology). Click here for file
