# Supplementary material for: Perioperative dynamic alterations in peripheral regulatory T and B cells in patients with hepatocellular carcinoma
Source: J Transl Med. 2012 Jan 25;10:14. doi: 10.1186/1479-5876-10-14 (PMC3292477; doi:10.1186/1479-5876-10-14)
Supplement: Additional file 1 — Table S1. Variables and point values used in DESS for liver cancer patient (History). [file 1479-5876-10-14-S1.DOCX]

## Table S1. Variables and point values used in DESS for liver cancer patient (History)

| **Variables** | **Points** | | | |
| --- | --- | --- | --- | --- |
|  | **0** | **1** | **2** | **4** |
| ***History*** |  |  |  |  |
| Age(Yrs) | ≤50 |  |  | >50 |
| Sex | Female |  |  | Male |
| Known liver lesions (Wks) | 0 | >0and≤3 | >3 and≤4 | >4 |
| HBV infection history(Yrs) | 0 | 1-10 | 11-20 | >20 |
| Increased AFP (Wks) | 0 | >0and≤2 | >2 and≤4 | >4 |
| Upper abdominal bleeding | - |  |  | + |
| Urine color change | - |  |  | + |
| Jaundice | - |  |  | + |
| Abdominal pain or distension | - |  |  | + |
| Constipation | - |  |  | + |
| Nausea or vomitting | - |  |  | + |
| Hematemesis | - |  |  | + |
| Hematochezia | - |  |  | + |
| Fatigue of lower extremity | - |  |  | + |
| Consciousness | Conscious | Hypersomia | Confusion | Coma |
| Fatigue | - |  |  | + |
| Appetite | Good | Average |  | Bad |
| Sleep | Good | Average |  | Bad |
| Stool and urine | Normal |  |  | Abnormal |
| Fever | - |  |  | + |
| Weight lost | - |  |  | + |
| Nutrition | Good | Median |  | Poor or Overweight |
| Edema of lower extremity | - |  |  | + |
| Smoking (Yrs) | Deny | ≤10 | 11-20 | >20 |
| Alcohol consuming (Yrs) | Deny | ≤10 | 11-20 | >20 |
| Hypertension (Yrs) | Deny | ≤5 | 5-10 | >10 |
| Diabetes mellitus(Yrs) | Deny | ≤5 | 5-10 | >10 |
| Chronic Hepatitis or Liver malignancy history of family | - |  |  | + |
