# Supplementary material for: Perioperative dynamic alterations in peripheral regulatory T and B cells in patients with hepatocellular carcinoma
Source: J Transl Med. 2012 Jan 25;10:14. doi: 10.1186/1479-5876-10-14 (PMC3292477; doi:10.1186/1479-5876-10-14)
Supplement: Additional file 2 — Table S2. Variables and point values used in DESS for liver cancer patient (Signs and physical exams). [file 1479-5876-10-14-S2.DOCX]

## Table S2. Variables and point values used in DESS for liver cancer patient (Signs and physical exams)

| **Variables** | **Points** | | | |
| --- | --- | --- | --- | --- |
|  | **0** | **1** | **2** | **4** |
| ***Signs and physical exams*** |  |  |  |  |
| Temperature(℃) | ≤37.3 | 37.4-38.1 | 38.2-39.1 | >39.1 |
| Heart rate(/minute) | 60-100 |  |  | >100,or<60 |
| Respiratory rate(/minute) | 16-18 | 19-20or12-15 | 21-24,or 8-11 | >24,or<8 |
| Blood pressure(mmHg) | <140/90 | SBP140-159 or DBP 90-99 | SBP160-179 or BP100-109 | SBP≥180or DBP≥110 |
| Abdominal tenderness or rebound tenderness | - |  |  | + |
| Enlargement of lymph nodes | - |  |  | + |
| Palpation of liver | - |  |  | + |
| Liver palms | - |  |  | + |
| Spider angioma | - |  |  | + |
| Heart&lung examination | - |  |  | + |
| Palpation of spleen | - |  |  | + |
| Murphy's sign | - |  |  | + |
| Intestinal gurgling sound(/ min) | - |  |  | + |
| Abdominal wall varicosis | - |  |  | + |
| Shifting dullness | - |  |  | + |

SBP: Systolic blood pressure DBP: Diastolic blood pressure
