# Supplementary material for: Perioperative dynamic alterations in peripheral regulatory T and B cells in patients with hepatocellular carcinoma
Source: J Transl Med. 2012 Jan 25;10:14. doi: 10.1186/1479-5876-10-14 (PMC3292477; doi:10.1186/1479-5876-10-14)
Supplement: Additional file 3 — Table S3. Variables and point values used in DESS for liver cancer patient (Laboratory test). [file 1479-5876-10-14-S3.DOCX]

## Table S3. Variables and point values used in DESS for liver cancer patient (Laboratory test)

| **Variables** | **Points** | | | |
| --- | --- | --- | --- | --- |
|  | **0** | **1** | **2** | **4** |
| ***Blood regular test*** |  |  |  |  |
| Hemoglobin(g/L) | Male120-160  Female110-150 | 90-lower limit of normal | 60-89 | <60 |
| HCT(%) | 37.0-50.0 |  |  | <37.0 or >50.0 |
| MCV (fl) | 79.0-101.0 |  |  | <79.0 or >101.0 |
| MCH( pg) | 26.0-35.0 |  |  | <26.0 or >35.0 |
| RDW (%) | 11.5-14.5 |  |  | <11.5 or >14.5 |
| WBC(×10^9^/L) | 4.0-10.0 | 2.5-3.9 |  | >10.0 or <2.5 |
| Neutrophil count (×10^9^/L) | 2.0-7.0 |  |  | <2.0 or >7.0 |
| Neutrophil(%) | 40.3-72.3 |  |  | <40.3 or >72.3 |
| Lymphocyte( %) | 20.4-51.0 |  |  | <20.4 or >51.0 |
| Lymphcyte count (×10^9^/L) | 0.8-4.0 |  |  | <0.8 or >4.0 |
| Monocyte(%) | 3.0-12.0 |  |  | <3.0 or >12.0 |
| Monocyte (×10^9^/L) | 0.1-0.5 |  |  | <0.1or >0.5 |
| Eosinophilic granulocyte(%) | 0.2-5.0 |  |  | <0.2 or >5.0 |
| Eosinophilic granulocyte (×10^9^/L) | 0.05-0.50 |  |  | <0.05 or >0.50 |
| Basophilic granulocyte(%) | 0-1.0 |  |  | >1.0 |
| Basophilic granulocyte (×10^9^/L) | 0-0.01 |  |  | >0.01 |
| Platelet(×10^9^/L) | 100-300 |  |  | <100 or >300 |
| MPV(fl) | 7.4-12.5 |  |  | <7.4 or >12.5 |
| PCT(%) | 0.108-0.282 |  |  | <0.108 or >0.282 |
| ***Liver and kidney function*** |  |  |  |  |
| Total Bilirubin (μmol/L) | 0-34.2 | 34.3-171.0 | 171.1-342.0 | >342.0 |
| Albumin (g/L) | 35.0-55.0 |  | 28.0-34.9 | <28.0 |
| Globin (g/L) | 20.0-35.0 |  |  | >35.0 |
| ALP(U/L) | 30-115 |  |  | >115 |
| γ-GT(U/L) | 0-54 | 55-124 | 125-263 | >263 |
| α-AFU(U/L) | 10-35 | 36-66 | 67-84 | >84 |
| GPDA(U/L) | 44-116 |  |  | >116 |
| Adenylate deaminase(U/L) | 0-18 | 19-30 | 31-35 | >35 |
| ALT (U/L) | 3-50 | 51-100 | 101-200 | >200 |
| AST (U/L) | 3-40 | 41-100 | 101-200 | >200 |
| Indirect bilirubin (μmol/L) | 3-14 |  |  | >14 |
| Direct bilirubin( μmol/L) | 1-7 |  |  | >7 |
| Cystatin C (mg/L) | 0.4-1.1 |  |  | >1.1 |
| A/G | 1.5-2.5 | 1.2-1.4 | 1.0-1.1 | <1.0 |
| Total Cholesterol(mmol/L) | 3.10-5.70 |  |  | <3.10 or >5.70 |
| Triglyceride(mmol/L) | 0.35-1.70 |  |  | < 0.35 or >1.70 |
| HDL(mmol/L) | 0.88-2.00 |  |  | <0.88 or >2.00 |
| LDL(mmol/L) | 0.67-3.37 |  |  | <0.67 or >3.37 |
| VLDL(mmol/L) | 0.31-1.25 |  |  | <0.31 or >1.25 |
| Na(mmol/L) | 135-145 |  |  | <135 or >145 |
| K(mmol/L) | 3.50-5.30 |  |  | <3.50 or >5.30 |
| Cl(mmol/L) | 96-108 |  |  | <96 or >108 |
| Ca(mmol/L) | 2.08-2.60 |  |  | <2.08 or >2.60 |
| P(mmol/L) | 0.81-1.62 |  |  | <0.81 or >1.62 |
| Fasting blood glucose(mmol/L) | 3.92-6.00 | 6.01-7.00 |  | >7.00 |
| ***Coagulation function*** |  |  |  |  |
| INR | 0.85 -1.15 | 1.16-1.70 | 1.71-2.20 | >2.20 |
| Fibrinogen (g/L) | 2.0-4.0 |  |  | <2.0 or >4.0 |
| APTT( s) | 22.0-36.0 | 36.1-40.0 |  | ≥41.0 |
| Thrombin time (s) | 14.5-21.5 | 21.6-24.4 |  | ≥24.5 |
| Prothrombin time prolonged( s) | 11.0~15.0 |  |  | >15.0 |
| D-dimer (μg/L) | 0-500 |  |  | >500 |
| ***Urine and stool regular test*** |  |  |  |  |
| Urine specific gravity | 1.000-1.030 |  |  | <1.000 or >1.030 |
| Urinary occult blood | - | ± | + | ≥++ |
| Urinary nitrite | - | ± | + | ≥++ |
| Urinary bacteria | 0-26.4 | 26.5-50.0 | 50.1-100.0 | >100.0 |
| Urinary turbidity | - | ± | + | ≥++ |
| Urinary protein | - | ±/+ | ++ | >++ |
| Urinary glucose | - | ± | + | ≥++ |
| Stool occult blood | - | ± |  | ≥+ |
| Urinary RBCs (/ul) | 0-22.7 | 22.8-50.0 | 50.1-100.0 | >100.0 |
| Urinary WBCs (/ul) | 0-13.2 | 13.3-90.0 | 90.1-170.0 | >170.0 |
| Urinary Cylinder(/ul) | 0-0.4 |  |  | >0.4 |
| Urinary Epithelial Cell(/ul) | 0-5.2 |  |  | >5.2 |
| Urobilinogen | - |  |  | ≥+ |
| PH | 4.5-8.0 |  |  | <4.5 or >8.0 |
| Stool color | Yellow | Brown |  | Black |
| ***HBV test*** |  |  |  |  |
| HSeAg (PEIU/ml) | 0-0.50 |  |  | >0.50 |
| HscAb (PEIU/ml) | 0-18.00 |  |  | >18.00 |
| HbsAg (ng/ml) | 0-0.50 |  |  | >0.50 |
| HBV DNA copy number | 0-5×10^3^ |  |  | >5×10^3^ |
| ***Tumor Marker*** |  |  |  |  |
| AFP (ng/ml) | 0-20.0 | 20.1-200.0 | 200.1-400.0 | >400.0 |
| CA125 (U/ml ) | 0-35.0 | 35.1-50.0 |  | >50.0 |
| CA153 (U/ml) | 0-28.0 | 28.1-35.0 | 35.1-50.0 | >50.0 |
| CA199 (U/ml) | 0-37.0 | 37.1-200.0 | 200.1-1000.0 | >1000.0 |
| Ferritin( ng/ml) | 7.0-323.0 | 323.1-400.0 | 400.1-500.0 | >500.0 |
| CEA (ng/ml) | 0-5.0 | 5.1-10.0 | 10.1-20.0 | >20.0 |
| Numbers of up-regulated Tumor markers | 0 | 1 | 2-3 | >3 |
