# Supplementary material for: Perioperative dynamic alterations in peripheral regulatory T and B cells in patients with hepatocellular carcinoma
Source: J Transl Med. 2012 Jan 25;10:14. doi: 10.1186/1479-5876-10-14 (PMC3292477; doi:10.1186/1479-5876-10-14)
Supplement: Additional file 4 — Table S4. Variables and point values used in DESS for liver cancer patient (Imaging). [file 1479-5876-10-14-S4.DOCX]

## Table S4. Variables and point values used in DESS for liver cancer patient (Imaging)

| **Variables** | **Points** | | | |
| --- | --- | --- | --- | --- |
|  | **0** | **1** | **2** | **4** |
| ***Imaging*** |  |  |  |  |
| Splenomegaly | - |  |  | + |
| Potal vein hypertension | - |  |  | + |
| Portal Vein Thrombosis(PVT) | - |  |  | + |
| Portal vein tumor thrombosis(PVTT) | - |  |  | + |
| Hepatic vein invasion | - |  |  | + |
| Inferior vena cava tumor thrombosis | - |  |  | + |
| Number of increased tumor markers | 1 | 2 | 3 | 4 |
| Liver occupying lesion size(cm) | 0 | >0,<3 | 3-5 | >5 |
| Chest benign findings | - |  |  | + |
| Liver cirrhosis | - |  |  | + |
| Hepatic abnormal perfusion | - |  |  | + |
| Ascitices | - | ± | + | ++ |
| Multiple Satellite nodules | - |  | + | ++ |
| Satellite nodules in different lobe | - |  |  | + |
| Tumor hemorrhage | - |  |  | + |
| Gallbladder benign lesions | - |  |  | + |
| Liver calcification | - |  |  | + |
| Liver cyst | - |  |  | + |
| Fatty liver | - |  |  | + |
| Hepatic hemangioma | - |  |  | + |
| Elevated FDG metabolization(PET) | - |  |  | + |
| Abdominal lymph node enlargement | - |  |  | + |
| Non-abdominal lymph node enlargement | - |  |  | + |
| Major vessel oppression | - |  |  | + |
| Distant metastasis | - |  |  | + |
