# Supplementary material for: Perioperative dynamic alterations in peripheral regulatory T and B cells in patients with hepatocellular carcinoma
Source: J Transl Med. 2012 Jan 25;10:14. doi: 10.1186/1479-5876-10-14 (PMC3292477; doi:10.1186/1479-5876-10-14)
Supplement: Additional file 5 — Table S5. Variables and point values used in DESS for liver cancer patient (Pathology). [file 1479-5876-10-14-S5.DOCX]

## Table S5. Variables and point values used in DESS for liver cancer patient (Pathology)

| **Variables** | **Points** | | | |
| --- | --- | --- | --- | --- |
|  | **0** | **1** | **2** | **4** |
| ***Pathology*** |  |  |  |  |
| Grade of Differentiation |  | I | II-III | IV |
| Pathologic type |  | Hepatocellular carcinoma |  | combination of hepatocellular &cholangiocarcinoma |
| Satellite | - |  |  | + |
| Lymph node metastasis | - |  |  | + |
| Tumor size(cm) | 0 | >0,<3 | 3-5 | >5 |
| Tumor number | 0 | 1 |  | >1 |
| Ascitices | - |  |  | + |
| Liver cirrhosis | - |  |  | + |
| Portal Vein Thrombosis(PVT) | - |  |  | + |
| Ill-defined mass | - |  |  | + |
| Gallbladder invasion | - |  |  | + |
| Tumor encapsulation | - |  |  | + |
| Gallbladder benign lesions | - |  |  | + |
| Major vessel oppression | - |  |  | + |
| Diaphragm adhesion | - |  |  | + |
| Tumor necrosis | - |  |  | + |
| Microvascular invasion | - |  |  | + |
| Portal vein tumor thrombosis(PVTT) | - |  |  | + |
| Bile duct invasion | - |  |  | + |
| Hepatic vein involvement | - |  |  | + |
| Cross segments or lobes tumors | - |  |  | + |
| Tumor hemorrhage | - |  |  | + |
